# A Secondary Analysis of the Prevalence and Clinical Profile of Psychiatric Referrals of Delirium in a Tertiary Care Hospital in Central India

**DOI:** 10.7759/cureus.74797

**Published:** 2024-11-29

**Authors:** Saloni Mishra, Varchasvi Mudgal, Rahul Mathur, Vijay Niranjan, Virendra Pal

**Affiliations:** 1 Psychiatry, Mahatma Gandhi Memorial (M.G.M) Medical College, Indore, IND

**Keywords:** central india, delirium, india, outcome, prevalence, tertiary care hospital, variables

## Abstract

Introduction: Delirium is a common and serious neuropsychiatric syndrome characterized by acute, fluctuating alterations in consciousness, cognition, and perception. It is associated with increased morbidity, mortality, length of hospital stays, and healthcare costs.

Aim: The aim of this study was to assess the prevalence and clinical profile of psychiatric referrals of delirium in a tertiary-care hospital.

Methods: A secondary analysis was conducted on patients with delirium referred to the CLP Unit, Department of Psychiatry, between June 2022 and June 2023. A comprehensive analysis was done using descriptive statistics to summarize the data, independent t-tests to compare group means, and chi-square tests to examine associations between categorical variables, providing a robust evaluation of the key relationships and patterns.

Results: The key findings indicate that the psychiatric referral rate was about 2.98% from various departments. The prevalence of delirium in our hospital was 13.2% of the total psychiatric referrals and about 0.4% of the total inpatients of the tertiary care hospital during the study duration. The mean age of delirium patients in the geriatric and non-geriatric groups was 71.6 years and 37.9 years, respectively. Most referrals were from the medicine department. Metabolic disorders and substance use were the most common physical and psychiatric comorbidities, respectively. Psychotropic use with supportive intervention was common (60.3%), with antipsychotics being the most commonly used class of psychotropics.

Conclusion: Delirium is a prevalent and clinically diverse syndrome that affects a significant proportion of hospitalized patients. It is a multidimensional disorder impacting a range of populations. Physical and psychiatric comorbidities play an important role in prognosis. While psychotropic use may be helpful symptomatically, it is not associated with better outcomes. Treating the underlying cause remains of paramount importance.

## Introduction

Delirium is a complex neuropsychiatric syndrome commonly encountered across various healthcare settings, often associated with prolonged hospitalization and increased mortality, which can be independent of age, prior cognitive functioning, and comorbidities [1,2]. It is a confusional state defined by an acute impairment of consciousness, often presenting with psychiatric symptoms [3]. The prevalence of delirium in hospitalized patients varies from 6-56% according to the Diagnostic and Statistical Manual of Mental Disorders, Fifth Edition (DSM-5) [4].

Overall, delirium rates between 24% and 42% have been reported in various hospital settings, with rates for comorbid organic disorders ranging from 9% to 23% [5-7]. Delirium often goes undetected and is misinterpreted in clinical settings [8-10]. Despite improved standardized screening tools, clinical supervision, and better interdisciplinary liaison, the diagnosis of delirium remains underreported [11]. The prevalence of delirium varies with the population being studied [12]. Prevalence rates for delirium among hospitalized adult patients range from 3% to 30% [13,14]. The consequences of delayed identification and treatment of delirium can be troublesome, including longer hospital stays, increased morbidity and mortality, and increased hospital costs and resource utilization [15].

According to available research, the prevalence of delirium in hospitalized patients in India is reported to be between 10% and 31% [16-18]. The study aims to assess the prevalence and clinical profile of delirium among adult psychiatric referrals in a tertiary care hospital. It seeks to evaluate the demographic characteristics of patients, including differences between geriatric and non-geriatric groups, and to identify common physical and psychiatric comorbidities associated with delirium. Additionally, the study investigates referral patterns across hospital departments, the use of psychotropic medications, and other interventions in delirium management. By analyzing associations between key variables, the study aims to enhance understanding of the condition's prevalence, contributing factors, and clinical outcomes, emphasizing the importance of treating underlying causes for better prognosis.

## Materials and methods

The present research was a secondary analysis conducted on patients with delirium who were referred to the Consultation-Liaison Psychiatry (CLP) unit in the Department of Psychiatry at MGM Medical College, Indore, Madhya Pradesh, between June 2022 and June 2023. The patient records were meticulously maintained by the CLP team within the Department of Psychiatry, ensuring that all psychiatric documentation was seamlessly integrated with patients’ overall medical care. The team documented detailed psychiatric assessments, diagnoses, and progress notes, creating a thorough record of each patient’s mental health treatment within the context of their broader medical needs. This approach facilitated continuity of care, as the records were made accessible to all healthcare professionals involved, allowing them to reference the latest psychiatric insights alongside other medical information. In this way, the CLP team’s diligent record-keeping played a crucial role in ensuring that patients received integrated, multidisciplinary care that addressed both their psychiatric and physical health needs.

The purposive sampling method was used. The inclusion criteria were adult patients (>18 years), either sex, with or without any existing psychiatric or physical comorbidity, who were admitted to any medical or surgical ward, intensive care unit, or emergency department and were diagnosed with delirium according to the DSM-5 criteria by trained psychiatrists of the CLP unit.

The exclusion criteria for the research were patients admitted to psychiatric wards of the hospital, patients with incomplete or missing records, and records of patients less than 18 years old.

Data on socio-demographic characteristics, medical history, medication use including psychotropic medicines, clinical course, delay to treatment, and confusion assessment method (CAM) was utilized for screening [19], and the outcome was extracted from the records using a structured proforma. A comprehensive analysis was conducted employing descriptive statistics using IBM SPSS Statistics for Windows, Version 17 (Released 2008; IBM Corp., Armonk, New York, United States) to summarize the data, independent t-tests to compare the means between groups, and chi-square tests to assess associations between categorical variables. This approach offered a strong evaluation of the main relationships and patterns within the data.

The research plan was approved by the institution's research ethics committee before the commencement of the study. The study used anonymized data from existing records and did not involve direct contact with patients or their relatives.

## Results

A total of 1276 psychiatric referrals out of the 42687 inpatients were observed during the study duration in the tertiary care center, bringing the psychiatric referral rate in our study to 2.98%. Out of the 1276 psychiatric referrals, 169 were delirium referrals (13.2%).

The mean age of younger adults in our study was 37.9±9.5 years, while the older population had a mean age of 71.6±6.5 years as mentioned in Table 1. There was a preponderance of male gender, 94 (55.6%); married patients, 87 (51.4%); employed patients, 98(57.9%); and patients belonging to urban localities, 107(63.3%).

**Table 1:** Socio-demographic profile of delirium referrals to the psychiatry department.

Variable	N	%
Younger adults (18-64 years)	113	66.8
Older adults (>64 years)	56	33.2
Gender		
Male	94	55.6
Female	75	44.4
Marital status		
Married	87	51.4
Unmarried	66	39
Divorced/separated	16	9.6
Employment		
Employed	98	57.9
Unemployed	71	42.1
Locality		
Rural	62	36.7
Urban	107	63.3
Total	169	100

Table 2 shows the clinical profile of delirium patients referred. The mean duration of stay for delirious patients was 9.4 ± 2.7 days while the mean CAM score used for screening on the day of referral was 4.3 ± 1.1. The mean duration of delay in psychiatric referral was around 2.3 ± 1.2 days.

**Table 2:** Clinical profile of delirium patients referred. CAM: Confusion assessment method

Parameter	Value
Mean duration of stay (days)	9.4 ± 2.7 days
The mean duration of delay in psychiatry referral	2.3 ± 1.2 days
Mean CAM Score	4.3 ± 1.1

In Table 3, it is seen that 73 (43.2%) patients were referred from the ICU, and others (96,56.8%) were from non-ICU settings/in-patient wards. In the context of the referring department, most of the referrals were from the Department of Medicine, 68(40.2%), followed by the Department of Surgery, 41 (24.2%) including their sub-specialties. Others like Orthopedics, Obstetrics, Oncology, and Respiratory Medicine made up the rest of the constitution.

**Table 3:** Clinical profile of delirium referrals to the psychiatric department.

Source of Referral	N	%
ICU	73	43.2
Non-ICU	96	56.8
Departmental Distribution		
Medicine	68	40.2
Surgery	41	24.2
Orthopedics	11	6.5
Obstetrics and Gynecology	19	11.2
Others	32	18.9
Total	169	100
Medical/Surgical diagnosis		
Metabolic disorders	37	21.8
Infection	35	20.7
Head injury	25	14.7
Drug-related	22	13
Stroke/paralysis	24	14.2
Liver disease	18	10.6
Others	22	12.9
Existing/Comorbid psychiatric diagnosis		
Schizophrenia and psychotic disorders	7	4.1
Depression	24	14.2
Bipolar disorder	13	7.6
Anxiety disorders	12	7.1
Dissociative disorders	5	2.9
Stress-related disorders	4	2.3
Substance use disorders	41	24.2
Others	8	4.7

Table 4 shows that the most common reason for referral for delirium patients was found to be abnormal or uncooperative behavior (68%), followed by a review for existing psychiatric illness (11.8%) and substance addiction-related management (10.6%). 

**Table 4:** Reason for referral in the context of delirium.

Reason for Referral	Percentage (%)
Abnormal or uncooperative behavior	68.0
Review for existing psychiatric illness	11.8
Substance addiction-related management	10.6

The most common medical or surgical comorbidity was found to be metabolic disorders, 37(21.8%), followed by infectious diseases of various origins 35(20.7%). Head injury, 25(14.7%); drug-related delirium, 22(13%); and stroke 24(14.2%) were other major contributors.

Substance use disorder was the most common comorbid psychiatric disorder in patients with delirium, 41(24.2%). In the context of other psychiatric disorders, depression, 24(14.2%); bipolar disorder, 13(7.6%), and anxiety disorders were also prominent, 12(7.1%). On excluding substance use disorders, around 40% of the total sample had some form of psychiatric comorbidity, since a single patient can present with more than one psychiatric disorder, the overall percentage is more than 40%.

Table 5 shows that the majority of the patients with delirium were provided with some form of psychotropic management, 102(60.3%) along with environmental and supportive intervention. Supportive and environmental interventions for delirium focused on minimizing distress, enhancing recovery, and addressing underlying factors. Environmental strategies included ensuring adequate lighting, orientation aids like clocks and calendars, reducing noise, and promoting a regular sleep-wake cycle. The use of sensory aids like glasses or hearing aids was also undertaken. Supportive interventions involved regular reorientation by staff, maintaining hydration and nutrition, managing physical discomfort such as pain, and encouraging early mobilization.

**Table 5:** Management profile of delirium patients.

Management	N	%
Psychotropic use with supportive intervention	102	60.3
Supportive and environmental measures	67	39.7
Psychotropic used	N	%
FGA - Haloperidol		
Oral	17	29.3
Parenteral	41	70.3
SGA		
Quetiapine	17	10.0
Olanzapine	12	7.1
Risperidone	9	5.3
Benzodiazepines	20	11.8
Mood stabilizers	15	8.8
Others	4	2.3
Outcome	N	%
Recovered/Improved	108	63.8
Worsened	14	8.4

On analyzing the outcome of delirium, it was found that most patients had either recovered or improved (63.8%) during their stay. Around a third of patients had an unchanged, worse, fatal, or unknown outcome, which was included in the worse outcome.

Out of the total 102 patients who received psychotropics, we observed that parenteral haloperidol was the most preferred psychotropic prescribed for delirium management followed by benzodiazepines, other antipsychotics, and mood stabilizers. Many patients were prescribed more than one psychotropic; thus, the total is more than 102.

There was no statistically significant result for the outcome of delirium whether supportive intervention was combined with psychotropics or not (Table 6).

**Table 6:** Test of significance in the usage of psychotropics in the context of improvement of delirium.

	Better outcome (recovered + improved)	Worse outcome	Chi-square	p-value
Psychotropic with supportive intervention	65	37	0.03	0.95
Only supportive intervention	43	24		

## Discussion

Delirium has always been a significant concern in hospital settings due to its burden on health, clinical outcomes, and significant prevalence, in addition to the economic burden added to the patient, as reported by Leslie et al. [20]. This study aimed to investigate the prevalence and clinical profile of delirium in a large sample of inpatients within a healthcare facility in central India, shedding light on various aspects such as referring departments, reasons for referral, medical and psychiatric diagnoses, management strategies, and outcomes at discharge to obtain a better understanding of the aftermath of delirium in healthcare.

The findings indicate that the psychiatric referral rate was about 2.98%, which concords with previous studies conducted in India [18]. The prevalence of delirium was found to be 169 (13.2%) of the total 1,276 psychiatric referrals and about 0.4% of total inpatients during the study duration. This finding is corroborated by Grover et al. [18], who found the prevalence rate of delirium to be between 0.28% and 0.53%. The lower prevalence of psychiatric referrals contrasts with previous similar studies, which reported a higher prevalence. This difference may be attributed to a lack of sensitization among ICU staff and the fact that many delirium referrals are redirected to the Department of Neurology [20]. In our study, the mean age of non-geriatric (18-64 years) delirium patients at the time of admission was 37.9 years, which also made up more than 65% of the sample size, while the mean age at admission for geriatric patients (>65 years) was 71.6 years. This suggests that delirium can occur across a wide age range and highlights the need for clinicians to remain vigilant for its signs and symptoms in diverse patient populations. Many earlier studies, including those by Khurana et al. and Rohatgi et al., have demonstrated similar mean ages for younger and older adults [21,22].

The study also identified an important aspect of the clinical profile regarding the type of referring, 73(43.2%) patients were referred from the ICU inclusive of mechanically ventilated patients and 96 (56.8%) were from non-ICU settings. In a previous study, the incidence of delirium in the ICU ranged from 45 to 87% which is dependent on various factors [16]. The medicine and surgery departments accounted for the highest number of delirium referrals (40.2% and 24.2% respectively). This finding aligns with previous research, as medical illnesses are a common precipitating factor for delirium [21]. However, it is noteworthy that delirium cases were also observed in other specialties as well. This highlights the importance of a multidisciplinary approach to the recognition and management of delirium.

The reasons for referral varied, with uncooperative/abnormal behavior and existing psychiatric illness being the most common. Similar findings were also reported by Grover et al. and Wilson et al. [18,23]. These findings underscore the need for healthcare providers to consider delirium in patients presenting with behavioral changes, even in the absence of a known psychiatric history. The delay in referral had a variety of reasons, stemming from a combination of lack of awareness, priorities, and systemic issues within the healthcare environment. First, ICU staff or other healthcare providers may lack the necessary training or awareness to promptly recognize early signs of delirium, often mistaking them for other conditions or viewing them as a temporary response to the ICU environment. This lack of sensitization causes delays in identifying and acting on the need for a psychiatric or neurological evaluation. Another contributing factor is the prioritization of immediate life-threatening conditions in ICU settings. In these high-stakes environments, where stabilization of physical health often takes precedence, symptoms of delirium can be deprioritized. Furthermore, unclear referral pathways lead to delays if there is ambiguity about whether the Department of Neurology or Psychiatry should handle delirium cases. This can result in staff uncertainty or back-and-forth between departments before a decision is made. There are also issues related to stigma and misconceptions surrounding mental health; some healthcare providers undervalue the importance of addressing delirium promptly or may delay referrals due to biases about mental health services. Resource limitations, such as staff shortages or heavy patient loads, can further exacerbate delays, particularly when access to specialists in Psychiatry or Neurology is limited. Lastly, bureaucratic and administrative hurdles in the referral process, including paperwork and procedural requirements, slow down timely interventions, especially in hospitals with complex administrative systems. Together, these factors contribute to delays in the referral process for delirium patients, potentially impacting the effectiveness of treatment and overall patient outcomes.

Understanding the medical and psychiatric diagnoses associated with delirium is essential for tailoring treatment and preventive strategies. In this study, we identified specific medical and surgical diagnoses frequently co-occurring with delirium. Multiple etiologies, metabolic factors, and infections were frontrunners for the etiology of delirium, in concordance with studies by Ormseth et al. [24], Panicker et al. [25], and Grover et al. [18]. This information can guide clinicians in identifying high-risk patients and implementing preventive measures.

This study examined pre-existing psychiatric diagnoses among delirium patients. Identifying a pre-existing psychiatric diagnosis is crucial, as these individuals may be at increased risk for delirium due to their underlying mental health condition. It was observed that patients diagnosed with delirium had around 40% comorbid psychiatric disorders (which excludes substance use disorders); previous literature has established an increased risk of delirium in patients with psychiatric disorders [26,27]. It was also found that substance use disorder, followed by mood disorders, was the most common comorbidity, similar to Panicker et al. [25], who demonstrated similar findings. These findings highlight the need for comprehensive psychiatric assessments in patients with known psychiatric illnesses to detect and manage delirium promptly, which is also supported by earlier studies by Van der Kuur et al. and Matoo et al. [26,27].

Effective management of delirium is crucial to improving patient outcomes. The first step in managing this potentially fatal condition is to identify delirium and address the underlying medical condition. It is essential to choose the right drug to treat perceptual, behavioral, and cognitive problems. Many studies have demonstrated the need for psychotropic use in delirium [28-30].

This study revealed that some delirium cases were managed with psychotropic medications in conjunction with supportive care, while others received only supportive care. Psychotropic medications play a role in managing symptoms such as agitation and hallucinations, but their use must be carefully considered due to potential side effects, especially in older adults.

Among the psychotropic medications used, parenteral haloperidol (70.3%) was the most commonly prescribed. Haloperidol has been a traditional choice for managing delirium, but there is growing recognition of the potential risks associated with its use, including extrapyramidal symptoms [31]. The study also noted the use of other second-generation and first-generation antipsychotics, benzodiazepines, and mood stabilizers, suggesting variability in clinical practice. Individualized treatment plans should consider the patient’s underlying conditions and the potential risks and benefits of psychotropic medications.

The outcomes of delirium at discharge provide valuable insights into the effectiveness of the management strategies. The study revealed a range of outcomes, better (recovery or improvement) or worse (worsening, and death). The proportion of patients with each outcome can inform healthcare providers about the challenges and successes in managing delirium cases.

It is important to note that delirium is associated with increased mortality, longer hospital stays, and higher healthcare costs [20]. Therefore, efforts to improve the recognition and management of delirium are critical for improving patient outcomes and optimizing healthcare resource utilization.

This study's key strengths include its large sample size and comprehensive analysis of delirium across multiple departments, providing valuable insights into its prevalence, clinical profile, and outcomes. The findings highlight the need for increased awareness and training among healthcare staff, especially in recognizing delirium early and improving referral processes. Identifying common comorbidities and medication practices offers guidance for more effective management, particularly in the use of psychotropic medications like haloperidol. The study underscores the importance of a multidisciplinary approach to care, with implications for developing standardized protocols to improve recognition, referral, and treatment of delirium, ultimately enhancing patient outcomes and optimizing healthcare resources.

However, this study had numerous limitations. Firstly, it was a secondary analysis, and the data may be subject to selection bias. Secondly, the study is limited to a single healthcare facility, which may limit the generalizability of the findings to other settings. Future research should explore delirium in diverse healthcare settings and populations. Additionally, the study did not delve into the specific dosages and durations of psychotropic medications used, which could provide insights into treatment patterns and potential side effects. The study also did not include the pediatric population due to inadequate data. Future studies could examine the impact of different psychotropic regimens on delirium outcomes.

## Conclusions

In conclusion, delirium is a common and diverse syndrome affecting many hospitalized patients. This study highlights delirium’s prevalence across departments, reasons for referral, referral delays, associated diagnoses, management strategies, and discharge outcomes. It emphasizes the need to train healthcare professionals to recognize symptoms early and promptly refer patients to psychiatry, improving care and reducing referral delays.

Awareness, early recognition, and tailored interventions are crucial to mitigate delirium’s impact on outcomes. The study also stresses the judicious use of psychotropic drugs and advocates a multidisciplinary approach and individualized care to address this complex syndrome effectively. Understanding delirium’s clinical profile can help healthcare providers optimize care and improve patient well-being.
